# Modulation of the immunological and neuroinflammatory microenvironment in older people with multiple sclerosis

**DOI:** 10.3389/fimmu.2026.1832582

**Published:** 2026-06-12

**Authors:** Celia Oreja-Guevara, Sven G. Meuth, Nikos Grigoriadis, Patrick Vermersch, Hans Peter Hartung, Per Soelberg Sörensen, Letizia Leocani, Klaus Schmierer, Ralf Gold, Óscar Fernández

**Affiliations:** 1 Department of Neurology, Hospital Clínico Universitario San Carlos, Instituto de Investigación Sanitaria del Hospital Clínico San Carlos (IdISSC), Madrid, Spain; 2 Department of Medicine, Faculty of Medicine, Complutense University of Madrid, Madrid, Spain; 3 Department of Neurology, University Hospital Münster, Münster, Germany; 4 Multiple Sclerosis Center, Second Department of Neurology, School of Medicine, Aristotle University of Thessaloniki, Thessaloniki, Greece; 5 Univ. Lille, Inserm U1172 LilNCog, Centre Hospitalier Universitaire (CHU) Lille, Fédération Hospitalo-Universitaire (FHU) Precise, Lille, France; 6 Department of Neurology, Medical Faculty, Heinrich-Heine-University, Düsseldorf, Germany; 7 Brain and Mind Center, University of Sydney, Sydney, NSW, Australia; 8 Department of Neurology, Palacky University Olomouc, Olomouc, Czechia; 9 Danish Multiple Sclerosis Center, Department of Neurology, Copenhagen University Hospital, Rigshospitalet, Copenhagen, Denmark; 10 Department of Clinical Medicine, Faculty of Health and Medical Sciences, University of Copenhagen, Copenhagen, Denmark; 11 Department of Neurology, Vita-Salute San Raffaele University, Milan, Italy; 12 Blizard Institute, Faculty of Medicine and Dentistry, Queen Mary University of London, London, United Kingdom; 13 Clinical Board Medicine (Neuroscience), The Royal London Hospital, Barts Health NHS Trust, London, United Kingdom; 14 Department of Neurology, Ruhr-University Bochum, St. Josef-Hospital, Bochum, Germany; 15 Department of Pharmacology, Faculty of Medicine, Institute of Biomedical Research of Malaga (IBIMA), Regional University Hospital of Malaga, University of Malaga, Malaga, Spain

**Keywords:** aging, immunosenescence, inflammaging, multiple sclerosis, neuroinflammation

## Abstract

Life expectancy and the age at onset of multiple sclerosis (MS) are increasing, and a growing proportion of people with MS (pwMS) are now older than 55–60 years. Aging modifies MS pathobiology, with the dominant disease mechanisms moving from focal, relapse-driven inflammation to chronic, compartmentalized neuroinflammation and neurodegeneration. In this narrative review, we summarize current knowledge on the relationship between brain aging and MS, integrating clinical, radiological, pathological and therapeutic evidence. We first discuss mechanisms of immunosenescence and “inflammaging”, including changes in adaptive and innate immunity, gut microbiota dysbiosis, mitochondrial dysfunction and blood–brain barrier dysfunction, and how these processes favor microglial activation, slowly expanding lesions, smouldering MS and progression independent of relapse activity. We then examine the impact of age on disability trajectories, cognitive decline and comorbidities, and the role of vascular and neurodegenerative mechanisms. The review also addresses age-related changes in safety and efficacy of disease-modifying therapies (DMT), with a focus on high-efficacy DMT, de-escalation and discontinuation strategies, and the management of infections, malignancies and polypharmacy in older pwMS. Finally, we describe new approaches relevant to this population, including Bruton’s tyrosine kinase inhibitors, neuroprotective and remyelinating agents, advanced cellular therapies and lifestyle-based interventions. We conclude by outlining practical implications for personalized treatment decisions in older pwMS and open questions that future clinical trials and biomarker studies must address.

## 1 Introduction

Multiple sclerosis (MS) is a chronic immune-mediated and neurodegenerative disease of the central nervous system (CNS). Approximately 2.9 million people worldwide are estimated to have MS (1, 2), with its prevalence increasing compared to previous decades (1, 3). MS used to be considered a disease of young adulthood. However, epidemiological data have shown a shift in MS prevalence toward later ages, with peak prevalence in the late fifties and early sixties (4). Although most patients are still diagnosed between the ages of 20 and 40, a recent study confirmed a rising age at onset of the first demyelinating event over the last three decades, with the average age at onset being 34 years (5). Also, the higher prevalence of older people with MS (pwMS) might be due to improvements in life expectancy. The incidence of late-onset MS (LOMS; when the onset of MS occurs at the age of 50 years or older) and very-late onset MS (VLOMS; when it occurs after 60) has increased (6, 7), likely reflecting improved diagnostic awareness, broader access to specialist care, and advances in neuroimaging. As pwMS age, the clinical phenotype changes. Older patients often present a lower relapsing activity and a more progressive form of the disease regardless of duration (8–10). Age-related changes in the immune system, such as immunosenescence and inflammaging (see section 3.1), together with an age-related increase in neurodegeneration and frequent comorbidities, may help explain the somewhat different disease course in older adults (11, 12). These changes and the higher prevalence of comorbidities and polypharmacy (13, 14) pose important challenges for managing older pwMS. Most clinical trials evaluating DMTs have excluded patients older than 55. Therefore, information on DMT efficacy and safety in this population is limited (15).

The modulation of the immunological and neuroinflammatory microenvironment in older pwMS is still incompletely understood, though recent work has clarified main mechanisms and identified possible therapeutic targets. In this review, we describe how age-related immune remodeling interacts with compartmentalized neuroinflammation and neurodegeneration in older pwMS, and how these changes influence disability progression, comorbidity burden, and treatment decisions. We address the consequences of aging for DMT efficacy and safety, de-escalation/discontinuation strategies, and new therapeutic approaches in a population that is still underrepresented in clinical trials. Finally, we discuss current unmet needs and priorities for future research and clinical care.

## 2 Immunosenescence

### 2.1 Age-related changes in the immune system

Immunosenescence refers to the age-associated remodeling of immune function, characterized not only by functional decline but also by qualitative changes toward a pro-inflammatory and dysregulated immune phenotype (16). The effects of immunosenescence vary across both the innate and adaptive immune system, resulting in qualitative and quantitative changes in different components of the immune system. Progressive thymic involution, reduced hematopoiesis, and alterations in immune cells, their microenvironment, and circulating factors are observed (16–19). Innate immune cells decline in function, characterized by impaired antigen processing and presentation capacity and effector mechanisms (20, 21). Natural killer (NK) cells, macrophages, monocytes, dendritic cells, and neutrophils change in terms of their responses and distribution (20–26). These changes include reduced reactive oxygen species (ROS) production in neutrophils and increased ROS levels in dendritic cells and macrophages; reduced migration, adhesion and chemotaxis in monocytes and macrophages; reduced cytokine secretion and phagocytosis by macrophages; changes in the number of NK cell subsets, cytotoxic activity and degranulation capacity (16, 27–32).

Regarding the aging adaptive immune system, the overall T cell population is maintained or even increased, but the relative number of certain subsets and the diversity of T cell receptor repertoire decreases (17). The number of naïve T cells, especially CD8+ T cells, in the periphery is reduced and its function is altered. Aged CD8+ T cells show features of exhaustion such as reduced CD28 expression, an increased number of antigen-experienced memory T cells with elevated CD57, killer cell lectin-like receptor subfamily G member 1 (KLRG1), T cell immunoreceptor with Ig and ITIM domains (TIGIT), and programmed cell death protein 1 (PD1) expression, higher levels of senescence-associated secretory phenotype (SASP), higher susceptibility to apoptosis, shortened telomeres, and upregulation of the immunoreceptor tyrosine-based inhibitory motif (ITIM) domain (16, 18–22, 33). CD4+ T cells have similarly altered activation with age, though to a lesser degree (23).

B cells also change with aging. Despite a relatively stable total number of B cells, there is reduced B cell receptor repertoire diversity, a lower number of naive B cells, an increased memory B cell population, and a higher proportion of the late/exhausted memory B cells subset, expressing higher levels of SASP markers including pro-inflammatory cytokines, inflammatory microRNAs, and cell cycle regulators (24).

Although the underlying causes of immunosenescence have not been completely elucidated, damage in genetic, epigenetic, cellular and molecular mechanisms have been observed (25, 26). In particular, age-associated epigenetic dysregulation (including altered DNA methylation patterns, histone modifications, and changes in noncoding RNA expression) may contribute to the functional decline of immune cells by disrupting gene regulation (27). For instance, in CD8+ T cells, aging is associated with a loss of chromatin accessibility at gene promoters, reduced binding of transcription factors such as nuclear respiratory factor 1 (NRF1), and decreased transcription of respiratory chain genes, which could explain the reduced oxidative phosphorylation capacity in older naïve T cells (33). These mechanisms may, at least partially, explain the increased susceptibility to infections, reduced response to vaccines, and a higher incidence of age-related diseases such as neurodegenerative, autoimmune, cardiovascular, and metabolic diseases, and cancer observed in older individuals (26).

### 2.2 Premature immunosenescence in MS

Premature immunosenescence has been observed in pwMS (28–30) and other neurodegenerative diseases (31). PwMS present early-onset thymic involution and alterations in T and B cell function compared to controls (28, 30, 32) (**Table 1**). Despite stable total naïve CD4 T-cell numbers, pwMS have lower total naïve CD4 signal joint T-cell receptor excision circles (sjTRECs). Younger pwMS present total naïve CD4 sjTREC numbers comparable to much older controls, especially patients with primary progressive MS (PPMS) (28). In line with these findings, alterations of inhibitory and costimulatory signaling within the CD8 T-cell compartment in young pwMS were comparable to changes in older controls. In particular, the decline in coinhibitory markers (KLRG1 and lymphocyte activation gene 3 [LAG3]) and the concomitant increase of the costimulatory molecule CD226 were more pronounced in young patients with PPMS than in patients with relapsing-remitting MS (RRMS), despite having a similar mean age (RRMS: 48 years; PPMS: 49 years; p=0.478) and time since first symptoms of MS (RRMS: 81 months; PPMS: 102 months; p = 0.379) suggesting accelerated immune aging as the disease progresses (30).

**Table 1:** Recent findings suggesting premature immunosenescence in patients with MS.

Reference	Participants (N)	Main results
Eschborn et al., 2021 (30)	*Cohort A (peripheral blood immunophenotyping):* RRMS (n=38) PPMS (n=40) Controls (n=40). *Cohort B (CSF immunophenotyping):* RRMS (n=51) PPMS (n=36) Controls (n=85). Groups in each cohort were subdivided into young (≤50 years) and old (>50 years) participants.	*Cohort A* *Premature immune aging in young patients with MS* Older pwMS had a more pronounced increase in intracellular expression of IFN-γ and TNF-α in T cells on stimulation (IFN-γ in CD4, p<0.01; IFN-γ in CD8, p<0.001; TNF-α in CD4, p<0.001; TNF-α in CD8; p<0.0001) than age-matched controls (NS; except TNF-α in CD8, p<0.05). Premature immune aging signatures of KLRG1, LAG3, CTLA-4, and CD226 were identified in younger pwMS (p<0.01). *RRMS vs PPMS* Age-dependent changes within the CD8 T-cell compartment were more pronounced in patients with PPMS versus patients with RRMS. *Cohort B* An age-dependent decrease in counts of lymphocytes, B and T cells (p<0.001), plasma cells and NK cells (p<0.01) were observed in patients with PPMS but not in patients with RRMS.
Zuroff et al., 2022 (32)	Untreated pwMS (n=40; mean age=43.3; range=18–72). Controls (n=49, mean age=43.3; range=20-84)	PwMS showed abnormal age-associated increases of activated HLA-DR+CD38+; p=0.013) and cytotoxic CD4 T cells, particularly in patients >60 (EOMES: P < 0.001). Aging MS patients failed to upregulate CTLA-4 expression on CD4 (p=0.014) and CD8 (p=0.009) T cells, coupled with age-associated increases in frequencies of B cells expressing costimulatory molecules.
Lorenzini et al., 2024 (34)	Untreated patients with RRMS (n=19; age range=21-53) Natalizumab-treated (n=12; age range=25-47) Anti-CD20–treated (n=10; age range=25-62) Controls (n=16; age range=22-53)	PwMS showed a lower frequency of Foxp3+ Tregs compared to controls (p=0.002). Younger pwMS had a lower frequency of t-Tregs compared to age-matched controls (p=0.039). Older patients displayed an increased frequency of CD45RA-GPA33+ t-Tregs in comparison with controls (p=0.033).
Hecker et al., 2021 (35)	RRMS (n=40; mean age=48.0; range=24-67) PPMS (n=40; mean age=48.0; range=26-68) Controls (n=60	Shorter leukocyte telomeres were detected in patients with RRMS than in patients PPMS and controls (p=0.003). Shorter telomeres at baseline were also associated with a higher conversion rate from RRMS to SPMS in the 10-year follow-up (p<0.05).
Yang et al., 2024 (36)	PwMS (n=52; ages 25-70) Controls (n=38; ages 23-65)	In patients over the age of 50, median p16INK4a levels were higher (0.25) compared to age-matched controls (0.12). Patientd in this age group undergoing B cell depletion therapy had reduced p16INK4a expression. p16INK4a expression was not associated MS disability outcomes.
Dema et al., 2025 (37)	EAE model 8-week- old mice (20 years old in humans) 40-week- old mice (50 years old in humans)	Aged mice exhibited more severe EAE, with significantly earlier neurological onset and greater disability accumulation (p<0.05). This was associated with a more inflammatory and neurodegenerative environment in the CNS, characterized by increased inflammatory infiltration (p<0.01), reactive microglia p<0.05), reactive astroglia (p<0.01), and axonal damage (p<0.0001). Age-related changes mainly affected adaptive immunity (altered T cell ratios (p<0.05), a pro-inflammatory Th1 response (p<0.05), highly suppressive Treg cells (p<0.0001), exhaustion of T cells (p < 0.01), altered B cell antigen presentation (p < 0.05), and reduced NK cell maturation and cytotoxicity (p<0.05). Transcriptomic analysis showed that fewer pathways and transcription factors were activated in aging mice, with a predominance of apoptosis-related, chromatin remodeling, and inflammatory pathways.
Picón et al., 2021 (38)	PwMS (n=263; median age=39.0; range=16–65) M- Patients (without LS-OCMB): (n=191; median age=40.0; range=16–65) M+ Patients (with LS-OCMB): (n=72; median age=35.0; range=18–62)	M- patients showed a significant decline in total lymphocytes, CD4+ and CD8+ T cells, and B cells (p<0.0001), NK cells (p<0.0002) and pro-inflammatory cytokine production (IFN-γ, TNF-α, GM-CSF, and IL-17) by B and T cells (p<0.02*), while monocytes remained unchanged. Reduced B cell TNF-α production was observed in both M+ and M- groups.
Hu et al., 2019 (39)	MS Patients: (n=18; median age=50.0; range = 28–74) Controls (n=105; median age=50.0; range = 23-86)	MS patients had significantly higher levels of TNF-α, IP-10, IL-10, and IL-8 compared to HC (p < 0.001). IP-10 and IL-8 increased more rapidly with age in MS (p <0.001).

CNS, central nervous system; EAE, experimental autoimmune encephalomyelitis; GM-CSF, Granulocyte/macrophage-colony stimulating factor; IFN-γ, interferon-gamma; LS-OCMB, lipid-specific oligoclonal IgM bands; NK, natural killer cells; NS, not significant; PPMS, primary progressive MS; RRMS, relapsing-remitting MS; TNF-α, tumor necrosis factor alpha; Tregs, regulatory T cells. *Different p values were reported for each cytokine and the most conservative p value has been included here.

A cross-sectional study that evaluated age-associated changes in circulating T cells in pwMS and controls showed that patients had abnormal age-related increases in activated CD4 T cells and cytotoxic CD4 T cells, particularly in patients over 60 years of age, and failed to upregulate CTLA-4 expression on both CD4 and CD8 T cells (32). Premature T cell senescence has been observed even in pediatric pwMS, who showed reduced circulating naïve T cells, signs of early thymic involution, and an increased proportion of memory T-cells, resembling the profile of 20- to 30-year-older controls (40).

In addition to earlier dysregulation of T and B cells and higher expression of cellular stress and pro-inflammatory markers (28, 30, 32, 34, 41), pwMS also have shorter telomeres in blood cells in comparison to healthy adults of similar age (28, 30, 32, 34) and in older pwMS compared to younger patients (42). Telomere attrition, a well-known predictor of morbidity and mortality in the general population (43, 44), may contribute to T and B cell dysfunction in MS by triggering DNA damage signaling and limiting the proliferation of immune cells, which is associated with premature cellular senescence (45). A systematic review revealed that shorter telomeres in pwMS are associated with higher disability, lower brain volume, increased relapse rate, and conversion from relapsing to progressive MS, independent of age (46). A recent study reported that associations between leukocyte telomere length and disease duration, disability severity, smoking, high-efficacy treatments, brain lesion volume and T2 lesion count were only observed in elderly, but not younger pwMS (42).

Metabolic alterations may also contribute to earlier immunosenescence in pwMS (47). PwMS show increased oxidative stress, dysregulated glycolysis and impaired mitochondrial function (see Section 3.1.1) (47). Mitochondrial activity in lymphocytes, which is lower in pwMS, is associated with disease severity (48).

In summary, evidence suggests that immunosenescence starts earlier in pwMS. One question that arises is when does immunosenescence appear in MS? A straightforward answer cannot be provided to this complex question since immunosenescence-related changes are more associated with biological age and immune microenvironment rather than chronological age. There is no single biomarker that can measure biological age (49); however some promising biomarker candidates have been identified (49–52).

**Figure 1:**
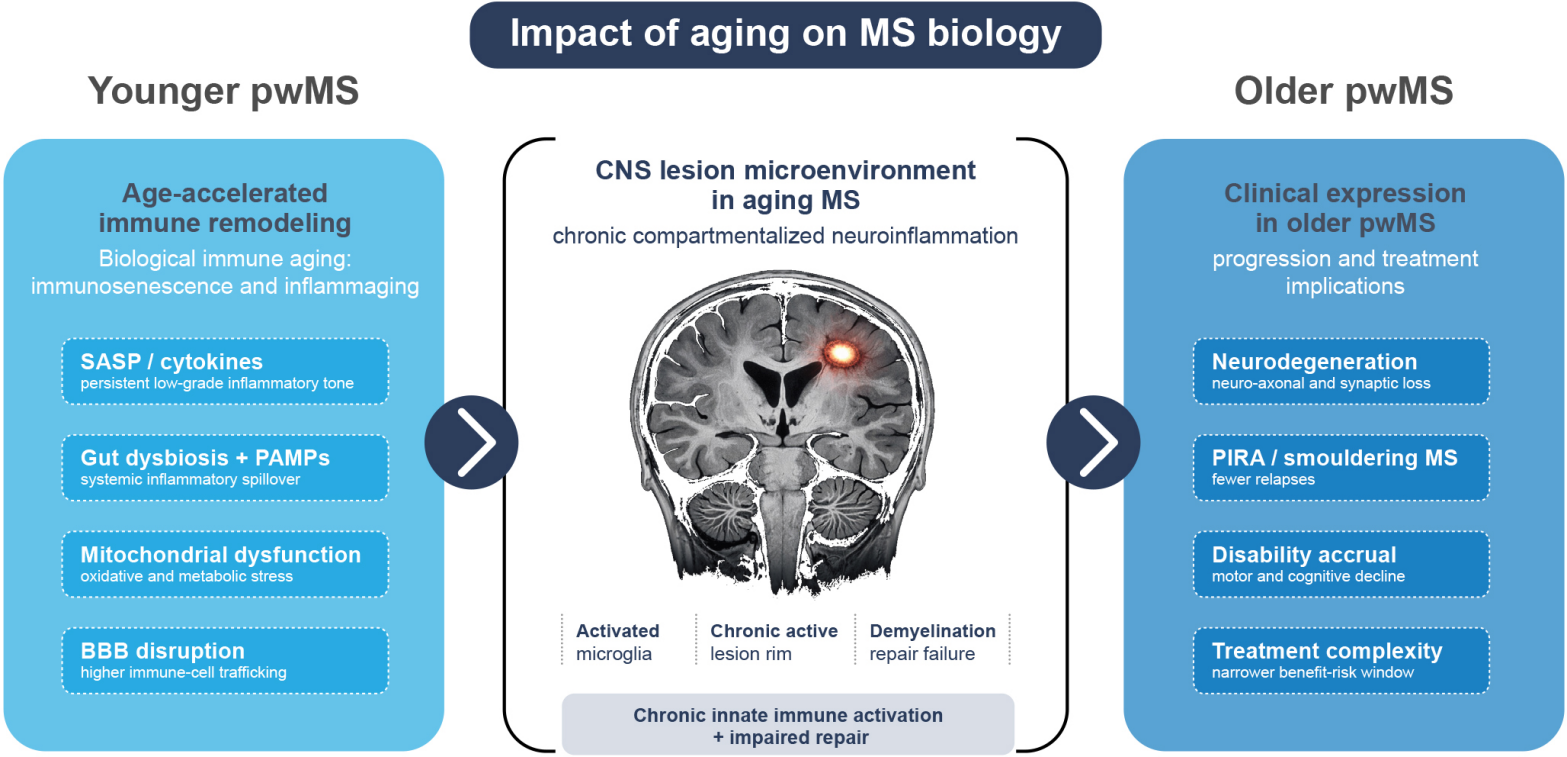
Biological changes in multiple sclerosis with aging. Schematic representation of how age-related biological processes may promote neuroinflammation and drive disease progression in multiple sclerosis (MS).

The evidence indicates that immune aging biomarkers in pwMS resemble those of much older reference populations (28, 30, 32, 34, 41). These results have also been found in patients with autoimmune diseases, such as systemic lupus erythematosus (53), psoriasis (54) and rheumatoid arthritis (55). More MS specific questions are, therefore, “how does MS accelerate biological immune aging?” and “which therapeutic interventions could be used to treat older pwMS to delay this premature aging process.” In the next sections, we will review recent evidence that provide some answers to these questions. The immune aging mechanisms described above are summarized in **Figure 1**.

## 3 Age-related pro-inflammatory changes and their systemic impact in MS

### 3.1 Inflammaging

Acute inflammation is a natural defense mechanism that facilitates immune cell recruitment to eliminate pathogens and promote tissue repair following injury or infection. However, when inflammation becomes chronic due to persistent exposure to the initial stimulus or dysregulation of signaling and effector pathways, it can cause harm (56). Inflammaging is a chronic, low-grade, inflammation that develops with aging even in the absence of acute infection or disease (57). This phenomenon is not only a consequence of increasing chronological age, but is also a marker of multimorbidity and mortality risk (56, 58). While centenarians and individuals who experience successful aging tend to present low levels of inflammation (59), older patients with diverse neurodegenerative and autoimmune diseases, including MS, have high levels of inflammation (**Figure 1**) (32, 60).

The causes of inflammaging are multifactorial. A key contributor is the SASP, characterized by secretion of pro-inflammatory cytokines, chemokines, and growth factors by senescent cells. The SASP causes an imbalance between the pro-inflammatory and anti-inflammatory immune responses, fostering chronic inflammation, and inducing senescence in otherwise normal cells (17, 61, 62). At the same time, chronic inflammation accelerates immunosenescence, impairing immune function and reducing the capacity to eliminate senescent cells and inflammatory mediators. This further exacerbates the cycle of inflammation and cellular aging in older individuals (63, 64). Within this context, the NOD-, LRR- and pyrin domain-containing protein 3 (NLRP3) inflammasome acts as an amplifier of SASP-related inflammation by sensing cellular stressors, such as mitochondrial dysfunction and oxidative damage. Its activation leads to the release of IL-1β and IL-18, perpetuating chronic inflammation. In older pwMS, persistent NLRP3 activation in microglia and astrocytes reinforces the inflammatory environment created by the SASP, contributing to disease progression (65). This process is further enhanced by cross-talk between pro-inflammatory B cells and myeloid cells, as secreted products from MS-implicated B cells induce pro-inflammatory cytokine expression (IL-12, IL-6, TNFα) in microglia and macrophages. This, in turn, propagates compartmentalized CNS inflammation, contributing to MS worsening (66).

Other factors contributing to inflammaging and immunosenescence in MS might be mitochondrial dysfunction and other metabolic alterations as well as gut microbiota dysregulation (48, 64, 67). **Table 2** displays several inflammaging markers in MS, integrating aging-related, inflammatory, and disease-specific biomarkers that have received attention in recent years.

**Table 2:** Potential biomarkers of inflammaging in MS.

Biomarker [ref]	Role
IDO1 (68, 69)	An enzyme that participates in: -tryptophan metabolism, which modifies the pathway from serotonin to kynurenine production, contributing to immune regulation and neuroinflammation in MS -maintaining chronic inflammation by modulating T-cell activity and neuronal excitability.
POM121L9P (70)	A pseudogene associated to neuroinflammation in MS, contributing to inflammaging by modulating immune response and cellular senescence
Activin A (38)	Elevated levels in CSF of aged MS patients may participate in neuroinflammation and immune cell dysregulation.
Calcium signaling dysregulation (71)	Imbalance in Ca2+/cAMP signaling contributes to neuroinflammation and neuronal aging in MS.
Circulating EVs (72)	EVs can display both anti- and pro-inflammatory roles on the innate and adaptive immune system, and are involved in several CNS functions.
GDF15 (73)	A biomarker of chronic inflammation in older pwMS
CSF cytokines: TNF-α, IL-6, IL-8, and IP-10 (39, 74–76)	Levels of pro-inflammatory cytokines in the CSF increase with age and serve as biomarkers of inflammaging. In patients with MS, an age-related change from Th1- to non-Th1-related cytokines has been observed.
Tryptophan metabolism (77)	Inflammaging redirects tryptophan metabolism toward the synthesis of kynurenine, an immunomodulatory catabolite. This initially adaptive anti-inflammatory response, can lead to imbalances in other tryptophan-derived metabolites
Sex-related inflammatory differences (e.g., IL-6, TNF-α, C-reactive protein) (78)	Sex-based differences in inflammatory mediators contribute to differential disease progression and severity in aging MS patients. The advantage in terms of immune system efficiency that women experience in the reproductive period is lost in the post-reproductive period.
miR-21 and miR-146a (79)	miRNAs target molecules belonging to the NF-κB/NLRP3 pathways. Circulating inflammamiRs, along with IL-6, can measure the degree of inflammaging.

CSF, cerebrospinal fluid; EVs, extracellular vesicles; GDF15, growth differentiation factor 15; IDO1, indoleamine 2,3-dioxygenase 1; IL, interleukin; POM121L9P, POM121 transmembrane nucleoporin like 9 pseudogene; TNF, Tumor necrosis factor.

#### 3.1.1 Mitochondrial dysfunction and other metabolic alterations

Mitochondrial dysfunction impairs oxidative phosphorylation, resulting in increased ROS production, oxidative stress, ATP depletion, and altered calcium homeostasis, which contribute to energy deficits in neurons and oligodendrocytes (80). Oxidative stress promotes neuroinflammation, myelin damage, and excitotoxicity (81), and is recognized as a common denominator in neurodegenerative disorders, including MS (81, 82). *In vivo* studies have demonstrated that brain concentrations of the antioxidant glutathione are significantly lower in patients with progressive MS compared to relapsing MS (RMS) and healthy controls, suggesting a higher involvement of oxidative stress in the progressive stage of MS (82).

Mitochondrial dysfunction in MS affects several CNS cell types. Post-mortem studies of pwMS showed axonal and neuronal mitochondrial respiratory dysfunction, such as reduced complex IV activity and mitochondrial DNA deletion–associated respiratory deficiency, that might contribute to neurological impairment and disease progression (83, 84). Also, there is a stage-dependent metabolic change in MS lesions, with reactive astrocytes showing a glycolytic/lactate-associated profile and demyelinated axons in chronic inactive lesions showing reduced glucose transporter type 3 (GLUT3) and monocarboxylate transporter 2 (MCT2) expression (85, 86). Experimental autoimmune encephalomyelitis (EAE) models have also shown that astrocytes respond to mitochondrial dysfunction by upregulating glycolysis, suggesting a cell type–specific metabolic adaptation (87). Additional evidence suggests that axonal mitochondrial size correlates with myelin sheath thickness (g-ratio) across species, with larger mitochondria present in axons with thinner myelin sheaths under homeostatic conditions and during advanced—but not early—remyelination (88). Following demyelination, the energy demands of axons increase, sparking production and anterograde transport of additional mitochondria from the neuronal soma to the demyelinated segment—a process termed the axonal response of mitochondria to demyelination (ARMD). However, this adaptive mechanism is delayed, leaving demyelinated axons transiently susceptible to metabolic stress and degeneration (89).

Peripheral immune cells in patients with MS, including T cells and monocytes, also have mitochondrial inefficiency and increased glycolysis (47). Mitochondrial and metabolic profiles of T cells appear to vary with disease stage and severity (48, 90). Patients with PPMS (mean age 59.8 years) showed lower mitochondrial mass, reduced oxidative phosphorylation (OXPHOS), and increased glycolytic metabolism compared to patients with secondary progressive MS (SPMS) (mean age 60.8 years) (90). These findings could reflect a stage-dependent change in T cell bioenergetics: in PPMS, T cells remain metabolically active, relying on glycolysis to compensate for mitochondrial inefficiency, whereas in SPMS, T cells may be transitioning into a more metabolically impaired state. Similar mitochondrial dysfunction has been observed in dermal fibroblasts from pwMS, which had a senescent phenotype characterized by reduced expression of mitochondrial proteins, diminished respiratory capacity, and mitochondrial depolarization and fragmentation (91). Environmental exposures also appear to modulate mitochondrial integrity in MS. Smoking and contact with pollutants such as heavy metals and pesticides accelerates mitochondrial damage by increasing ROS production and impairing mitochondrial DNA repair (92–94). These observations suggest that reducing environmental toxin exposure may help preserve mitochondrial function and slow disease progression in aging individuals with MS.

As mitochondrial function declines with age, the accumulation of mitochondrial DNA (mtDNA) mutations increases (82). Mitochondria are not only bioenergetic hubs but also an important source of damage-associated molecular patterns (DAMPs) (95), including mtDNA and mitochondrial proteins. The interplay between DAMPs and inflammaging creates a vicious cycle, where ongoing inflammation leads to further cellular damage, releasing more pro-inflammatory DAMPs and perpetuating inflammaging and neurodegeneration (47, 96, 97).

Recent studies have also highlighted the role of microRNAs (miRNAs) in modulating inflammaging, particularly a subset known as inflammamiRs that target NF-κB/NLRP3 inflammatory pathways (79). These miRNAs can be encapsulated in extracellular vesicles (EVs), such as exosomes, facilitating cell-to-cell communication and modulating inflammatory pathways in recipient cells (89). This nano-scale regulation of inflammaging adds an additional layer of complexity to MS progression.

In addition to mitochondrial dysfunctions, a relationship between age, disability, and metabolic changes has been observed in pwMS (98, 99). In one study, higher disability (Expanded Disability Status Scale, EDSS ≥3) was associated with older age, elevated levels of homocysteine, uric acid, LDL-cholesterol, advanced oxidized protein products, and a higher prevalence of metabolic syndrome, while HDL-cholesterol was lower. The LDL/HDL ratio, homocysteine concentration, and presence of metabolic syndrome emerged as metabolic predictors of disability (98). However, as the study did not directly compare metabolic alterations in older and younger patients, it remains unclear whether disability, rather than age alone, could also cause metabolic alterations.

#### 3.1.2 Gut microbiota dysregulations and neuroinflammation

Aging is accompanied by changes in the gut microbiota, characterized by reduced diversity and functional imbalance—a state referred to as dysbiosis—which is recognized as a contributor to systemic inflammation (100–102). Age-related increases in gastrointestinal permeability facilitate the release of microbial-derived, immunostimulatory molecules (i.e. pathogen-associated molecular patterns [PAMPs]), into the circulation, some of which penetrate the CNS (103). These microbial products, including lipopolysaccharides and peptidoglycans, can activate peripheral immune cells and can contribute to inflammatory processes (104, 105).

The role of alterations in the gut microbiota as a factor contributing to the pathophysiology of MS remains under investigation. Dysbiosis in aging could amplify neuroinflammatory processes, although direct evidence linking age-associated microbiota changes with neuroinflammation in MS remains limited. In aging, there is a decline in beneficial commensal species and an expansion of pro-inflammatory bacterial populations, leading to dysbiosis (106). This microbial imbalance may disrupt the gut-blood-brain barrier, allowing inflammatory mediators to reach the CNS, where they can trigger or worsen neuroinflammation (107).

Microbial metabolites such as short-chain fatty acids (SCFAs), produced by fermentation of dietary fiber, participate in the regulation of immune function (108). In aged MS patients, reduced SCFA production due to dysbiosis may impair anti-inflammatory processes, increasing susceptibility to neuroinflammatory damage (109, 110). Specific microbial signatures have been associated with MS progression. For instance, patients with progressive MS had elevated *Enterobacteriaceae* and *Clostridium g24 FCEY*, and decreased *Blautia* and *Agathobaculu*m; several *Clostridium* species were associated with higher EDSS and fatigue scores (111). Interestingly, the role of *Akkermansia* in MS remains controversial. Although associated with MS pathology in some human studies (112), EAE models have suggested a potential protective role, as the presence of this bacterium correlated with lower disability and inflammation (111). In line with these findings, another study revealed that patients with progressive MS had a higher microbial imbalance compared to RMS (i.e. more decrease in *Faecalibacterium prausnitzii*, a SCFAs-producing bacteria, and larger increase in *Ruthenibacterium lactatiformans, Hungatella hathewayi*, and *Eisenbergiella tayi)* (113). The decrease in *Faecalibacterium prausnitzii* was associated with a higher Multiple Sclerosis Severity Score (MSSS) in progressive MS (113).

Beyond immunomodulation, the gut microbiota also plays a role in regulating iron homeostasis. Ferroptosis—a regulated form of iron-dependent cell death characterized by lipid peroxidation—has gained attention in the context of aging and MS (114). Aging-related dysregulation of iron metabolism might influence the progression of MS​ (114–116). Dysbiotic oral bacteria can also release bacterial products into the bloodstream and cross the blood-brain barrier (BBB), promoting inflammation and further altering gut–brain axis homeostasis (117).

Targeted interventions aiming to restore gut microbial balance may offer therapeutic potential in MS, particularly in older patients. Strategies such as dietary modulation, probiotics, and fecal microbiota transplantation are being explored for their capacity to attenuate neuroinflammation (118, 119). Indeed, distinct gut microbiomes have been consistently found in longevous populations, which could inform interventions to improve late-life health (120).

### 3.2 Role of aging in blood-brain barrier dysfunction

The BBB is a highly selective membrane that regulates the exchange of molecules between the bloodstream and the CNS. The BBB maintains homeostasis by restricting the entry of harmful substances and immune cells. In MS, however, the compromised BBB allows the infiltration of peripheral immune cells, antibodies, and pathogens into the CNS, causing and amplifying inflammatory responses which lead to demyelination and neuronal damage (121).

With advancing age, the permeability of the BBB increases (122). Changes include disorganization of tight junction proteins, increased permeability, and impaired function of efflux transporters such as P-glycoprotein, which normally prevents toxin accumulation (123–125). Some of the potential causes for BBB dysfunction in older pwMS include decreased cerebral blood flow, increased EVs release, endothelial dysfunction, inflammaging, and lower vascular density (72, 126). These age-related changes sensitize brain-resident myeloid cells to systemic pro-inflammatory signals, such as microbial-derived PAMPs, resulting in altered microglial cytokine responses (127).

The increase of BBB permeability with age may be associated with a higher risk of neurodegenerative diseases. With aging, most inflammatory cells inflict remote damage by releasing soluble substances that diffuse into the surrounding tissues, triggering the activation of microglia and astrocytes (128). Chronic inflammation has been associated with increased BBB permeability and cognitive decline (60, 129). In SPMS, microglial activation is elevated compared to RRMS and healthy controls (130). Moreover, age seems to selectively intensify microglial activation in individuals with MS, a pattern not observed in healthy aging (130). When microglia are altered, the clearance of myelin debris is compromised, which in turn compromises remyelination and contributes to the progressive loss of myelin integrity (131).

In PPMS, patients show fewer gadolinium-enhancing lesions on MRI (132, 133). However, studies examining tight junction protein expression in post-mortem brain tissue have demonstrated abnormalities of zonula occludens-1 (ZO-1), a structural component of the BBB endothelium, in active white matter lesions and normal-appearing white matter in PPMS with frequencies comparable to those observed in relapsing and SPMS, and abnormalities in chronic white matter plaques more frequently in PPMS than in SPMS, suggesting that in PPMS the endothelial dysfunction is not transient but rather a persistent, smouldering process (134). This chronic, low-grade BBB dysfunction is consistent with the neuropathological concept of slowly expanding lesions, a hallmark of PPMS, in which ongoing CNS-compartmentalized inflammation drives progressive axonal loss and disability accumulation independently of new focal inflammatory activity (135).

### 3.3 Epstein-Barr virus, aging, and disease progression

The Epstein-Barr virus (EBV) is a human herpesvirus that persists in latent form in B cells throughout life after initial infection. Compelling epidemiological evidence indicates that EBV infection precedes the onset of MS, with a 32-fold increase of MS risk among EBV-infected individuals (136). A large biobank study confirmed this association, showing that prior EBV exposure can be detected up to 15 years before diagnosis and is strongly predictive of MS risk (137). There is also some evidence suggesting an association between EBV and the risk of dementia, though this risk appears less pronounced than for herpes simplex virus type 1 (HSV-1) infection (137, 138). Although the exact pathophysiology remains unknown, these findings indicate that infections with common viruses may contribute to the risk of MS and other neurodegenerative disorders.

Immunosenescence and inflammaging may accentuate EBV-driven neuroinflammation and MS progression by weakening antiviral immune surveillance and sustaining a pro-inflammatory immune environment. Studies have reported that older pwMS have higher EBV antibody titers compared to younger patients (139). Furthermore, T cells from EBV- and cytomegalovirus-positive individuals have higher levels of several senescence-associated markers, including KLRG1, an increase in the fraction of terminally differentiated T cells, and a decrease in the diversity of the T-cell repertoire (140). Also, preliminary evidence suggests that EBV may contribute to progression independent of relapse activity (PIRA), although these findings require further research (141).

One proposed mechanism through which EBV may contribute to MS pathogenesis involves molecular mimicry. Antibodies targeting the EBV nuclear antigen (EBNA_386-405_) cross-react with GlialCAM_370-389_, a CNS protein. A study that analyzed 12,708 EBV-seropositive MS patients and healthy controls showed that a combination of host genetic predispositions and viral variants defines the individual risk for MS, with certain combinations associated with an up to 260-fold increased risk. In healthy individuals with high EBNA-1 antibodies, NKG2C+ and NKG2D+ NK cells eliminated stressed autoreactive GlialCAM-specific cells. In contrast, MS patients had impaired NK cell responses and lower levels of these specific NK cell subsets (142). These findings suggest that EBV-related molecular mimicry may become pathogenic when antiviral and autoreactive-cell clearance mechanisms are insufficient.

In addition to its well-documented latency in B cells (143), EBV has been identified in astrocytes and microglia within MS lesions (144). Although further studies are needed, glial EBV infection could represent a potential contributor to neurodegeneration and MS progression. EBV-associated exosomes have also emerged as potential mediators of intercellular communication and viral pathogenesis. These EVs can carry viral DNA, RNA, proteins, and metabolites that support EBV persistence and immune modulation (145). PwMS, specifically those with active disease, display increased expression of EBV proteins in serum-derived exosomes. These EBV-positive exosomes induce an inflammatory response in monocyte-derived macrophages, leading to increased secretion of pro-inflammatory cytokines and chemokines (146). The specific effects of aging on EBV-driven exosomal pathways remain unexplored and warrant further investigation; these mechanisms may be relevant in older pwMS, in whom immunosenescence and inflammaging could favor persistence of EBV-related inflammatory signals and compartmentalized CNS inflammation.

## 4 Clinical impact of aging on MS

### 4.1 Comorbidities

Older pwMS experience additional age-related challenges, including the natural decline in physical and cognitive function, and a rising burden of comorbid conditions that can affect their disease progression and overall health (10, 147). Most old pwMS have at least one comorbidity (148), with the most common being cardiovascular and metabolic diseases (hypertension, dyslipidemia, obesity, and diabetes), psychiatric disorders (depression and anxiety), endocrine and autoimmune diseases (thyroid disorders), and musculoskeletal conditions (osteoporosis and chronic pain) (10, 149).

The presence of multiple comorbidities is further associated with increased disability and mortality in elderly patients (13, 147). In a retrospective study, patients without comorbidities had a median EDSS score of 3.5, while those with three or four comorbidities had a median EDSS score of 6; after correction for sex and age at diagnosis, the risk of reaching EDSS 6 rose with every additional comorbidity by 22% (10). Dementia, epilepsy, and psychiatric comorbidities were the comorbidities most strongly associated with severe disability (10). Although vascular comorbidities have been suggested as being associated with accelerated disability worsening (150) this association did not remain significant after adjusting for sex and age at diagnosis in more recent studies (10).

Frailty, smoking, and obesity should also be considered when assessing factors that may contribute to progression in older pwMS. In a prospective multicenter study of patients with PMS, frailty was more frequent in patients with late severe PMS and was associated with higher EDSS scores, as well as inflammatory and metabolic vulnerability (151). Smoking is a modifiable factor associated with progression. In a cohort study of patients with relapsing-remitting onset, ever-smokers had a higher risk of conversion to SPMS than never-smokers (152). A recent registry suggested that smoking and obesity can interact to worsen disability progression and cognitive performance in pwMS (153).

### 4.2 Sex hormones

Age-related changes in sex hormones might be one of the factors interacting with the MS course. However, the exact influence of hormonal changes in the disease course is not clear. In women, menopause, on one hand, has been found to accelerate worsening of the Multiple Sclerosis Functional Composite (MSFC) (154), but, on the other hand, it was associated with stable EDSS (155) or even decelerated worsening in the EDSS (154), and it was not associated with 6-month confirmed disability progression or SPMS (156). Regarding rates of relapse, some systematic reviews concluded that there is no difference before and after menopause (157), while others (with meta-analysis) stated that menopause can be associated with a reduction in the relapse rate (158, 159). In men, andropause could be associated with physical and cognitive worsening (160), and small trials have suggested that testosterone supplementation may slow brain atrophy and improve cognition in RRMS (161).

### 4.3 Disease progression

Relapses in pwMS are less frequent with advancing age (162). However, when relapses in older patients do occur, they tend to be more severe and have reduced likelihood of recovery, and are associated with an increased risk of disability progression independent of relapses (PIRA) (10, 163–165). Older pwMS accumulate disability at a faster rate compared to younger counterparts with similar disease duration. For instance, one study showed that patients with adult-onset MS (AOMS) reached an EDSS score of 7 after a median of 19.1 years, whereas those with LOMS and VLOMS reached the same disability milestone after 13.4 and 9.7 years, respectively (10).

With aging, not only do motor impairment and gait instability worsen, but cognitive impairment also becomes more evident (166). However, the specific impact of age on different cognitive domains remains uncertain. Some studies suggest that the most commonly impaired domains in older patients are the same as in young patients, i.e. information processing speed, followed by verbal learning, executive function, and visuospatial learning (166), whereas other studies indicate that older patients have higher impairments in episodic memory, verbal fluency, and planning (167).

These discrepancies may, at least in part, be influenced by the age at MS onset. Patients with LOMS have shown more disability and cognitive impairments in visual memory and working memory than patients with AOMS, which is in line with the hypothesis that MS diagnosed later in life progresses faster (168, 169). In LOMS, lower neuron densities are observed in the thalamus and pons, together with lower numbers of actively demyelinating lesions and a higher degree of compartmentalized inflammation than in AOMS (169). Post mortem evidence suggests a dissociation of neuronal loss from demyelination in advanced MS (170), a finding confirmed and expanded to a lack of association with compartmentalized inflammation and disability in patients with LOMS (169). Also, comorbidities may increase the progression of MS when diagnosed later in life, with one study reporting that in LOMS having at least one vascular risk factor was associated with slower information-processing speed (assessed by the Symbol Digit Modalities Test, SDMT) and poorer social cognition (assessed by Reading the Mind in the Eyes Test, RMET) (171).

Disability accrual in older pwMS appears to depend on a complex interaction between age-related neurodegenerative changes and disease-specific pathophysiology. In the general population, brain volume loss occurs at an annual rate of up to 0.5% after the age of 50–60 (172). In contrast, MS-related neurodegeneration follows a different trajectory characterized by accelerated atrophy from early disease stages (173, 174). Subcortical structures are more affected in MS, with the thalamus presenting the highest degree of atrophy, while cortical atrophy becomes more apparent in later stages of MS (175). There is also substantial synaptic damage in MS, with synaptic loss in the spinal cord and loss of dendritic spines in cortical gray matter, which may occur partly independently of demyelination and axonal loss and may contribute to disability accrual (176, 177). The combination of MS-specific neurodegeneration and age-related brain atrophy results in a phenomenon where the brains of individuals with MS appear older than their chronological age. The “brain-age” paradigm, which uses neuroimaging data to estimate biological brain age, has revealed that a higher brain-predicted age difference (brain-PAD) at baseline is associated with disability worsening (178). In addition, the brain-predicted disease duration gap has been proposed as a complementary measure to differentiate MS-related neurodegeneration from normal aging (179). Even if aging influences the pattern of MS-related brain atrophy, it does not appear to worsen the neurodegenerative processes characteristic of MS. Although ventricular enlargement and whole-brain atrophy are consistently observed across all ages in MS, the rate of whole-brain atrophy does not accelerate as a direct consequence of aging (173). In fact, studies have shown that the highest rate of neurodegeneration occurs in younger patients during the period of most active immune inflammation (174).

Demyelination and remyelination processes are also affected by age. Older age is associated with impaired recruitment of oligodendrocyte precursor cells (OPCs) to demyelinated lesions and their limited differentiation into oligodendrocytes, leading to inefficient myelin repair (180). Furthermore, aging-related increases in inflammatory activity from microglial cells and astrocytes, as well as mitochondrial dysfunction, increased oxidative stress and higher expression of senescence-associated genes and markers, further compromise tissue integrity (31, 181, 182). In particular, cellular senescence in progenitor cells within demyelinated lesions contributes to remyelination failure in progressive MS, partly through the secretion of high-mobility group box 1 (HMGB1), which inhibits oligodendrocyte differentiation (183). Both demyelination and neurodegeneration processes could be also worsened by impairments in the glymphatic system function and by the presence of cerebral small vessel disease observed with aging (184).

Chronic active lesions with iron deposition, known as paramagnetic rim lesions (PRL), have been identified as predictors of disability progression and brain atrophy (185, 186). However, the effect of PRLs seems to diminish with age. Their prevalence decreases in older patients, and those with longer disease duration have fewer PRL (187). Also, a retrospective study showed that the proportion of PRL decreased as lesion age increased (188).

### 4.4 Efficacy of disease-modifying treatments

Limited research has been published on the efficacy of DMTs in older pwMS since the last review articles addressing this topic (15, 189). Briefly, meta-analyses have shown that the efficacy of DMTs in older pwMS declines with advancing age. A meta-analysis that included over 28,000 MS patients demonstrated that DMT efficacy in preventing disability progression decreases with age, with no predicted benefit beyond 53 years (190). Similarly, the efficacy of DMTs was higher in younger than older patients in both reduction of the annualized relapse rate (ARR) and disability progression (191). However, another meta-analysis found that DMTs reduced clinical and radiological activity regardless of age (192).

Only a few randomized controlled trials have evaluated treatment efficacy in patients older than 55 years (193–196). And even these studies excluded patients older than 60 or 65 years old, despite patients older than 60 representing approximately one-quarter of the MS population (197). The available data suggest that the benefit–risk balance of DMTs may change with age, supporting the need for individualized treatment decisions. Evidence regarding DMT de-escalation and discontinuation in older pwMS is discussed in section 5.4 Pharmacological considerations and safety.

It is well-known that the risk of adverse events increases with age, mainly due to immunosenescence and the higher prevalence of comorbidities. However, not all DMT are associated with safety concerns in older patients. Some DMTs, like dimethyl fumarate, resulted in a higher incidence of lymphopenia in older patients (198), whereas others, such as cladribine and teriflunomide did not (199, 200). Also, B-cell depletion treatments (i.e. rituximab) have been associated with increased infection risk that is not modulated by previous DMTs or the duration of treatment (201).

Due to the lack of evidence of the effectiveness and safety of DMTs in older adults, patients with LOMS are less commonly treated with DMTs in general, and with high-efficacy therapy (HET) in particular (202–204). There is a need for larger studies assessing the long-term risk-benefit profile of DMTs in aging MS populations and potential treatment de-escalation.

## 5 Therapeutic strategies for older patients with MS

### 5.1 Immunomodulatory and neuroprotective interventions

The majority of older pwMS present a degree of progression (205). Although several potential treatments for progressive MS have been assessed in recent years, few of them have obtained positive results and approval. One of these treatments has been siponimod, which is approved for SPMS with active disease. The approval was based on the results of the EXPAND trial that showed a reduction of 21% of the confirmed disability progression, with the subgroup of patients with active SPMS mainly contributing to these findings (mean age of active SPMS patients: 46.6 years; non-active SPMS: 49.5) (193, 206).

Treatments for non-active SPMS remain an unmet need, particularly in older pwMS in whom conventional anti-inflammatory therapies are often insufficient to halt disability accumulation. One promising therapeutic approach is Bruton’s tyrosine kinase (BTK) inhibition, which is suggested to modulate both B-cell function and compartmentalized immune activity mediated by microglia and macrophages (207, 208). However, recent clinical trial results indicate that the efficacy of BTK inhibitors may differ across molecules and MS phenotypes. The phase 3, event-driven HERCULES trial in non-relapsing SPMS showed that tolebrutinib significantly delayed 6-month confirmed disability progression compared with placebo, with the observed benefit predominantly attributable to PIRA (209). By contrast, preliminary results from the phase 3 PERSEUS trial in PPMS showed no difference in delaying 6-month composite confirmed disability progression between tolebrutinib and placebo, although tolebrutinib significantly reduced new and enlarging brain lesions and slowed brain volume loss on MRI (210).

Other BTK inhibitors are also being explored across the MS spectrum. Evobrutinib, which initially showed proof-of-concept evidence in a phase 2 trial, failed to demonstrate superiority over teriflunomide in two phase 3 studies in RMS (evolutionRMS1 and evolutionRMS2) (211). Fenebrutinib has recently met primary endpoints of reducing relapse rates (versus teriflunomide) in RMS (FENhance) and showing non-inferiority to ocrelizumab in slowing disability progression in PPMS (FENtrepid) (212). However, most trials evaluating BTK inhibitors (BIIB091 [NCT05798520], evobrutinib (1^211^), orelabrutinib [NCT04711148], remibrutinib [NCT05147220] and fenebrutinib [NCT04586023; NCT04544449]) have not included individuals above 55 years, limiting the evidence on their efficacy and safety in older populations.

Chimeric antigen receptor T-cell (CAR-T) cell therapy consists of collecting a patient’s T cells, genetically engineering them to express a chimeric antigen receptor directed against a specific target, and reinfusing them to selectively eliminate or modulate pathogenic immune-cell populations. CAR-T cell therapies, which have revolutionized the field of hematologic malignancies, are now being explored in patients with progressive MS. Several clinical trials that included pwMS above 55 years of age are presented in **Table 3**. The ability of CAR-T cells to gain intrathecal access makes them a promising option for treating pathologies within the CNS (217). Results of the first use of CD19 CAR-T cells (KYV-101) in two patients (36 and 47 years old) with MS showed tolerable short-term safety. CAR-T cell presence and expansion in the cerebrospinal fluid (CSF), without clinical signs of neurotoxicity, and intrathecal antibody reduction were observed (218).

**Table 3:** Clinical trials evaluating treatments that included patients older than 55.

Treatment name (type)	Years of age; diagnosis	Clinical trial name, phase – status (reference)	^a^Primary endpoint or ^b^primary result if available (comparator)
Ibudilast (phosphodiesterase inhibitor and macrophage migration inhibitory factor modulator)	21-65; PPMS or SPMS	SPRINT-MS, phase 2 - Completed (213)	53% of the patients in the ibudilast group had primary progressive disease (vs 52% of those in the placebo group)
		Subanalysis Ehrardt et al., 2023 (214)	^b^GCIPL atrophy was 79% slower in the ibudilast group, mainly driven by the PPMS cohort (vs placebo)
		Subanalysis Nakamura et al., 2024 (215)	^b^Slower rates of overall brain atrophy with ibudilast (vs placebo)
Masitinib (tyrosine kinase inhibitor)	18-65; PPMS, non-active SPMS	MAXIMS, phase 3 – ongoing (NCT05441488)	Time to confirmed progression assessed by the EDSS at 96 w (vs placebo)
Tolebrutinib (BTK inhibitor)	18-60; non-active SPMS	HERCULES, phase 3 – completed (209)	22.6% of patients in the tolebrutinib group had 6-m CDP (vs. 30.7% in the placebo group)
Fenebrutinib (BTK inhibitor)	18-65 (46–65 in Germany and Italy); PPMS	FENtrepid, phase 3 – ongoing (NCT04544449)	Time to onset of composite 12-w CDP (vs ocrelizumab)
IMU-838 vidofludimus calcium (inhibitor of dihydroorotate dehydrogenase)	18-65; SPMS (active or non-active) and PPMS	CALLIPER, phase 2 – ongoing (NCT05054140)	Annualized rate of percent brain volume change during treatment period (up to 120 w) (vs placebo)
SpironolacTOne (aldosterone receptor antagonist) and famciclovir (antiviral agent)	NA	NA	NA
MSC-NP (mesenchymal stem cell-neural progenitor therapy)	18-65; PPMS or SPMS	STOP-MS, phase 2 – completed (NCT03355365)	^b^No statistically significant difference in EDSS Plus was found between the MSC-NP and placebo. Exploratory subgroup analysis demonstrated that in patients with EDSS 6.0-6.5 there was a significantly higher percentage of improvement in T25FW and 6MWT in the MSC-NP group (vs placebo)
KYV-101 (CD19 CAR-T cell therapy)	25-70; refractory PPMS or SPMS	NA, phase 1 – ongoing (NCT06451159)	Presence of CAR-T cells in CSF; incidence and severity of AEs and DLT
	18-65; non-active and PMS	NA, phase 1 – ongoing (NCT06138132)	Frequency of DLT at each dose level
	18-60; refractory PPMS or SPMS	KYSA-7, phase 2 – ongoing (NCT06384976)	CDP on the EDSS at least 12 w vs anti-CD20 mAb
Rapcabtagene autoleucel - YTB323 (CAR-T cell therapy)	18-60; non-active SPMS	NA, phase 1/2 – ongoing (NCT06675864)	Number of participants with AEs and SAEs
CC-97540 (immunomodulatory small molecule therapy)	18-60; RMS, PMS	Breakfree-2, Phase 1 – ongoing (NCT06220201)	Number of participants with AEs, SAEs, DLT and other safety outcomes
Simvastatin (statin)	18-65; SPMS	MS-OPT, phase 2 – completed (NCT03896217)	Effect on cerebral blood flow over 20 w (vs placebo)
	25-65; SPMS	MS-STAT2, phase 3 – completed (NCT03387670)	Time to CDP assessed by EDSS every 6 m up to 54 m (vs placebo)
Metformin as add-on treatment (AMPK activator and metabolic modulator)	18-70; PPMS or SPMS	MACSiMiSE-BRAIN, phase 2 – ongoing (NCT05893225)	Change in walking speed as measured by the T25FW at 96 w (vs placebo)
Metformin (AMPK activator and metabolic modulator) and lipoic acid (cofactor for mitochondrial enzymes)	25-70; PPMS or SPMS	OCTOPUS, multi-stage trial – ongoing (EUDRACT 2021-003034-37)	Whole brain atrophy rate (SOC vs placebo; SOC+lipoic acid; SOC+metformin)
Foralumab (human anti-CD3 monoclonal antibody)	18-75; non-active SPMS	NA, phase 2 – ongoing (NCT06292923)	Change from baseline for [18F]PBR06-PET scans for microglial activation at 12 w (foralumab 50 μg, foralumab 100 μg, placebo)
Hydroxychloroquine (antimalarial and immunomodulatory) and indapamide (thiazide-like diuretic)	18-60; SPMS	NA, phase 2 - unknown	Change in T25FW performance between the 6 m and 18 m visit
Adrenocorticotropic hormone (pituitary-derived peptide hormone)	≥18; SPMS, PPMS, or PRMS	NA, phase 2 - overdue	Proportion of patients with a 20% worsening in T25FW at 36 m (vs placebo)
Currently approved DMTs			
Siponimod (S1P receptor modulator)	18-60; active SPMS	NA, phase 2-3 – completed (NCT04925557)	Change from baseline in PET activation of PBR06^1^ at 12 m (vs ocrelizumab)
Cladribine (purine antimetabolite and selective immune reconstitution therapy)	30-65; SPMS	CLASP-MS, phase 2-3 – ongoing (NCT05961644)	Percent brain volume to total intracranial (vs placebo) volume at 26 and 122 w (vs placebo)
	≥18; PMS	ChariotMS, phase 2-3 – ongoing (NCT04695080)	The 9-HPT peg speed (tasks/second) at 24 m; 9-HPT proportion of patients who do not deteriorate at 24 m (vs placebo)
	21-65; SPMS	NA, phase 4, – ongoing (NCT04550455)	Time to achieve NEDA-4 at 96 w (vs baseline) Decrease of serum/CSF NfL at 48 w (vs baseline)
Ocrelizumab (humanized monoclonal antibody targeting CD20)	18-65; PMS	CONSONANCE, phase 3 – ongoing (NCT03523858)	Proportion of Participants with NEP (from bl to 96 w, from 96 w to 192 w, from bl to 192 w) Proportion of Participants with NEPAD (from bl to 96 w, from 96 w to 192 w, from bl to 192 w)
Currently approved supplements			
Lipoic acid (cofactor for mitochondrial enzymes)	18-70; PMS	LAMPS, phase 2 – completed (NCT03161028)	Change in mobility assessed by T25FW at 24 m (vs placebo)
N-acetyl cysteine (precursor of glutathione)	40-70; PPMS or SPMS	NACPMS, phase 2 – ongoing (NCT05122559)	Progression of brain, thalamic and cervical cord atrophy at 15 m. Safety and tolerability at 15 m (vs placebo)
Nicotinamide riboside (precursor of nicotinamide adenine dinucleotide)	18-65; PPMS or SPMS	Norseman, phase 2 – ongoing (NCT05740722)	Proportion of patients with sustained disability progression assessed by EDSS, T25W or 9HPT up to 30 m (vs placebo)
Bile acid (metabolic regulators & gut-brain signaling molecules)		NA, phase 1-2 – completed (NCT03423121)	AEs did not significantly differ vs placebo (216).
Behavioral treatment			
Cognitive reserve-building activities	18-65; RRMS, SPMS or PPMS	CRAMS, phase 2, – ongoing (NCT05691192)	Change in score on the SDMT at 12 and w (vs passive control) Change in score on the MSNQ at 12 and w (vs passive control)

6MWT, 6-minute walk test; 9HPT, 9-hole-peg test; AEs, adverse events; AMPK, AMP-activated protein kinase; bl, baseline; BTK, Bruton’s Tyrosine Kinase; CAR-T, Chimeric Antigen Receptor T-Cell; CDP, Confirmed disability Progression; CSF, cerebrospinal fluid; DLT, dose-limiting toxicities; GCIPL, Ganglion cell + inner plexiform layer; m, months; mAb, monoclonal antibody; MSNQ, Multiple Sclerosis Neuropsychological Questionnaire; MSC-NP, mesenchymal stem cell-neural progenitor; NA, not available; NEDA, no evidence of disease activity; NEP, no evidence of progression; NEPAD, no evidence of progression and no active disease; NfL, neurofilaments; PET, positron emission tomography; PPMS, primary progressive MS; S1P, sphingosine 1 phosphate; SAE, serious adverse event; SDMT, Symbol Digit Modalities Test; SOC, standard of care; SPMS, secondary progressive MS; T25FW, Timed 25-Foot Walk; w, weeks.

^1^ PBR06 is a second-generation PET radioligand targeting the 18-kd translocator protein to assess microglial activation.

A study with *in vivo* visualization of PRL evaluating the associations between PRL with disability progression and brain atrophy has shown that greater PRL disappearance rates were associated with reduced rates of confirmed disability progression and new PRL were associated with increased rates of PIRA (185). These findings highlight the potential of treatments targeting microglia-mediated brain inflammation, such as foralumab or siponimod. Other pharmacological approaches under investigation include remyelination therapies, neuroprotective agents, and strategies targeting age-related neurodegeneration, such as senolytic drugs and mitochondrial support therapies (**Table 3**). On the other hand, minocycline has shown efficacy in reducing glial fibrillary acidic protein (GFAP) and minocycline modulates matrix metalloproteinases (MMPs) in people with clinically isolated syndrome (CIS) (219), although minocycline did not add any beneficial effects when added to interferon-beta in RRMS (220). Further research is needed to determine minocycline safety and efficacy in older pwMS.

### 5.2 Remyelination treatments

To date, clinical trials assessing pharmacological remyelination in MS have reported mixed or negative results, and no agent has yet demonstrated sufficient efficacy for regulatory approval (221, 222). Clemastine fumarate showed modest improvements in visual evoked potential latency without short-term clinical benefit (223), and several remyelinating antibodies or small molecules have failed in phase 2–3 trials, possibly due to their lack of access to the CNS (221, 222). Ibudilast, initially developed as a phosphodiesterase inhibitor, emerged as a potential neuroprotective therapy in progressive MS, with phase 2 data and additional analyses suggesting beneficial effects on slowly enlarging lesions, brain atrophy and retinal thinning (213, 215). However, no phase 3 trials have been conducted in pwMS. Also, the M1 muscarinic receptor antagonist PIPE-307, which showed remyelinating effects in preclinical models, failed to meet primary and secondary efficacy endpoints in a phase 2 trial in RMS (224). Combination strategies such as metformin plus clemastine (CCMR Two) have demonstrated improvements in visual evoked potential latency, but again without short-term clinical benefit (225). Overall, these studies highlight the translational gap between biological remyelination and meaningful disability outcomes, particularly in older patients with progressive disease. Interestingly, a recent systematic review found that glatiramer acetate, teriflunomide, fingolimod, siponimod, ponesimod, and alemtuzumab influenced oligodendrocyte lineage cells in cell cultures or histological analysis *in vivo*, suggesting a remyelination effect (222). However, it remains unknown whether these effects are also observed in older adults, who are, on the other hand, the ideal target for remyelination treatments.

### 5.3 Promising treatments from preclinical research

EVs are being explored as experimental therapeutic tools and drug-delivery platforms, but their application in MS remains at an early, preclinical stage. EVs are small structures that are released in the extracellular space by all types of cells and carry many molecules, including lipids, DNA, RNA, and proteins. EVs, often divided into exosomes and microvesicles, can cross the BBB, making them potential carriers for targeted drug delivery in neurodegenerative conditions such as MS​ (226). They have the potential to improve regeneration in different tissues after damage (227). Also, EVs derived from mesenchymal stem cells (MSCs) could have neuroprotective properties and counteract age-related decline in CNS function​ by modulating immunosenescence, oxidative stress, inflammaging, telomere dysfunction, autophagy inefficiency and metabolic dysregulation (227). A recent study in mice has demonstrated that human embryonic stem cell-derived exosomes (hESC-Exos) can reverse cellular senescence by restoring proliferative capacity in senescent cells, leading to rejuvenation in aging mice, as evidenced by extended lifespan, improved physical performance, and reduced aging markers. The key mechanism involved exosomal miR-302b targeting cell cycle inhibitors Cdkn1a and Ccng2, reversed proliferative arrest without safety concerns over 24 months, highlighting its potential as a therapeutic strategy for pathologies in which senescence is involved (228). Clinical studies have not been conducted yet.

Recently, a novel glia-enriched stem cell 3D model was found to recapitulate the neuroinflammatory and neurodegenerative characteristics of MS (229). This platform could be relevant for identifying treatments for older pwMS, whose disease is compartmentalized in the CNS with potentially less pronounced effects on inflammation and neurodegeneration (190, 230). The organoid model mimics the chronic innate inflammation that occurs with older age in MS (231) by integrating neurons, astrocytes, oligodendrocytes, and microglia into a functional system that responds to inflamed CSF from MS patients, highlighting the vulnerability of oligodendrocytes to inflammation (229). By enabling the high-throughput screening of neuroprotective and remyelinating therapies, this model offers a preclinical platform for testing novel interventions aimed at halting neurodegeneration and promoting repair in aging MS patients.

Another potential treatment aimed at eliminating dysregulated microglia in MS is navitoclax (ABT-263), a small molecule BCL-2 family inhibitor and senolytic drug. By targeting anti-apoptotic proteins such as BCL-2, BCL-xL (BCL2L1), and BCL-w, navitoclax disrupts cell survival mechanisms, leading to the clearance of senescent and proinflammatory cells. In a study in EAE, navitoclax reduced inflammation, improved motor function and neuronal survival, and decreased white matter damage (232). These findings suggest that senolytic therapy could be a promising strategy for modulating neuroinflammation and slowing MS progression.

### 5.4 Pharmacological considerations and safety

Age-related physiological changes can alter the pharmacokinetics and pharmacodynamics of DMTs, impacting absorption, distribution, metabolism, and excretion. These changes may influence both treatment efficacy and safety, making responses to DMTs somewhat less predictable in older patients. Pharmacokinetic changes during aging include decreased renal and hepatic clearance, an increased volume of distribution for lipid-soluble drugs, and prolonged drug half-life, which may require dose adjustment for renally excreted DMTs (233). Moreover, pharmacodynamic alterations in older adults include increased sensitivity to various drug classes, such as immunosuppressants, anticoagulants, psychotropics, and antidiabetics (233). The cumulative effect of these changes and the higher risk of adverse events, such as progressive multifocal leukoencephalopathy (PML) with age (234), highlights the need for careful DMT selection and close monitoring to balance therapeutic efficacy and safety in older MS patients.

The potential impact of MS and DMTs on cancer risk has been a subject of debate, with studies reporting contradictory results (235). A recent retrospective study conducted in France that included 140,649 MS cases found that people with MS had a slightly increased overall cancer risk compared to the general population, particularly women (235). The risk varied by cancer type, being lower for prostate, breast, and colorectal cancers but higher for bladder, brain, and cervical cancers. Cancer incidence decreased in patients aged 65 and older, probably due to lower participation in national cancer screening programs—leading to underdiagnosis—or competing mortality risks (235). In contrast, a retrospective study in Canada of 14,313 MS cases reported that cancer risk in pwMS was no different from that of the general population (236). They observed an age-dependent effect of DMTs on cancer risk, with a higher risk in those aged 62 or older and a potential protective effect at younger ages. The increased risk of cancer in older pwMS exposed to DMTs could be due to longer treatment durations, age-related immune senescence, or the inherently higher baseline cancer risk in aging populations. Further studies are needed to understand the influence of age and DMT exposure on cancer risk.

Despite most of the current clinical trials including patients older than 55 years, only a few include patients over 70. An exception is the ChariotMS study, which was co-designed with pwMS and no upper age limit (237). The evaluation of therapeutic strategies for pwMS should be done by randomized controlled trials that include at least one-third of participants being older than 55, reflecting the demographic distribution of the MS population (197). More patient-reported outcomes (PROs) and clinical assessments other than the EDSS, such as the nine-hole-peg-test (9HPT) focusing on meaningful functions for pwMS with limited or no ambulation should be used to evaluate treatment outcomes in this population (238–240). Both the 9HPT and PROs can provide additional cost-efficient, and remotely accessible information on disability progression in a standardized and structured manner (197), even in older pwMS.

Concerns about the safety of DMTs in older pwMS, together with the age-related decline in focal inflammatory activity and the higher burden of comorbidities, have resulted in growing interest in DMT de-escalation and discontinuation strategies (225, 241). Observational cohorts suggest that de-escalating from higher efficacy to lower efficacy DMTs can result in a lack of disease stability, with younger age and inflammatory disease activity prior to de-escalation being risk factors for inflammatory disease activity post de-escalation (242). An observational study including patients older than 50 with nonactive MS explored whether discontinuing HET was associated with an increased risk of relapse compared with continuing HET. The study showed an increased risk of relapse after discontinuing HETs that affect immune cell trafficking (i.e. natalizumab and fingolimod), but there was no significant increase in risk after discontinuing HET that deplete B-cells (i.e. anti-CD20 therapy) (203). Another real-world cohort study suggested that HET provided less benefit with aging, supporting a de-escalation approach to balance efficacy and safety in older pwMS (204).

Predicting which patients might benefit from DMT de-escalating or discontinuation is difficult, and the use of tools or scores could help. In fact, the VIAADISC scores predicted the risk of disease reactivation after discontinuation from dimethylfumarate and teriflunomide, showing that the discontinuation was safe in pwMS above age 45–55 and with long-lasting stability; the study also showed that the risk after de-escalation from natalizumab and fingolimod was too high and should be avoided by lateral switch (242).

In the randomized DOT-MS trial, discontinuation of first-line DMT after ≥5 years of stability led to disease reactivation (mainly radiological activity) in 17.8% of patients versus none in those who continued treatment, highlighting that DMT discontinuation is not risk-free even in long-term stable disease (243). Data from the DISCOMS trial that included patients aged ≥55 years with no recent relapse or new MRI activity could not conclude whether DMT discontinuation is non-inferior to continuation (244). In this study, patients who discontinued DMT had a modest risk of recurrent (subclinical) disease activity, but the extension study reported no relapses in both the continuation and discontinuation groups, with low rates of new MRI lesions (245). Taken together, these findings suggest that discontinuing DMT at 55 years of age might be appropriate in some cases (245), but has also risks of disease reactivation in other cases (246). When considering de-escalation or discontinuation, the decision must be individualized and based on prior disease activity, duration of disease stability, exposure to HET, comorbidities and patient preferences.

### 5.5 Lifestyle modifications

Exercise is increasingly recognized as a promising approach for maintaining function in MS and aging populations (247). Regular physical activity has been shown to reduce autoimmune responses by limiting peripheral immune cell infiltration into the CNS, preventing T cell senescence and inflammaging, reducing circulating inflammation, and promoting an anti-inflammatory state (226–228). Additionally, exercise increases neuroplasticity, preserves brain volume, and supports the BBB, reducing oxidative stress and delaying neurodegeneration (229, 230). Exercise can also result in beneficial epigenetic modifications (231). For older adults with MS, exercise plays a key role in maintaining mobility, balance, and cognitive function, having positive effects on fatigue and quality of life (QoL) (232, 233). Despite these benefits, older individuals tend to have more sedentary lifestyle behaviors (234), emphasizing the need for structured, accessible programs to maximize its therapeutic potential in aging MS populations (235). Results from ongoing clinical trials testing the effect of exercise on brain atrophy and physical function specifically in older pwMS will provide more details on the benefits of these interventions on the disease progression (236, 237). **Table 4** displays systematic reviews and meta-analysis on the effect of physical activity in patients with MS.

**Table 4:** The effect of physical exercise in patients with MS: systematic reviews and meta-analysis.

Study objective [reference]	Mean age range (years)	Conclusion
To explore the effect of exercise on cognitive function (248)	29.24- 64.3	Exercise significantly improved cognitive function in middle-aged and elderly patients*. However, exercise had no significant effect on improving cognitive function in young patients. Subgroup analysis demonstrated that the older the age, the greater the effect on cognition.
To assess the effects of exercise on balance, walking ability, walking endurance, fatigue, and QoL (249)	16.3-61.6	Exercise improves balance, walking ability, walking endurance, fatigue, and QoL in patients with MS. Resistance exercise and aerobic exercise were the most effective interventions for improving fatigue and quality of life, respectively. A younger age* was associated with a larger improvement in fatigue.
To evaluate if exercise interventions affect disease activity and progression (250)	18-71	Cross-sectional studies suggest the beneficial effects of high physical activity or fitness levels on clinical outcomes; however, these findings are not supported by high-quality evidence.
To determine the effects of resistance training interventions on walking and muscle strength in middle-aged¥ (251)	NR (mean ± SD: 49.69 ± 9.26)	Resistance training significantly improves muscle strength, but has modest and non-significant improvements in gait. Studies combining resistance training with motor control exercises provide results of greater clinical significance in terms of gait. Resistance training has variable positive effects on fatigue and the quality of life.
To analyze the effect of different types of exercise on muscular fitness and cardiorespiratory fitness (252)	31.3- 65.1	Combined (aerobic and resistance) and resistance training seem to be the most effective exercises to improve muscular fitness and aerobic exercise for cardiorespiratory fitness in people with MS.

* young: <45 years old; and middle-aged and older adult: ≥45 years old); ¥middle age: 45–64 years, advanced age: >65 years, and middle age + advanced age: >45 years.

NR, not reported; QoL, quality of life; SD, standard deviation.

On the other hand, dietary interventions may help reduce symptom severity, improve cognition, and maintain QoL in people with MS (253–257). However, the effect on disability is still inconclusive. A meta-analysis of RCTs found no significant influence of diet on EDSS (mean age: 40 years) (254). In contrast, an observational study in older pwMS reported that adherence to the Mediterranean diet was independently associated with a lower prevalence of disability, cognitive impairment, symptom severity, depression, anxiety, perceived stress, poor sleep quality, and physical inactivity (253). Prior studies showed similar results with vegetable and fish intake, which are part of the Mediterranean diet, predicting lower EDSS scores (258). A recent systematic review of observational studies in MS has concluded that there is some evidence associating diet with disability and QoL, but the effects on other MS-related outcomes are less clear and need further prospective research (259).

The Mediterranean diet has also been related to reduced markers of immunosenescence, including lower accumulation of terminally differentiated memory CD8^+^ T cells (260). Other dietary approaches, such as the Paleolithic, low-carbohydrate and low-fat diets, have shown potential benefits in reducing neuroinflammation and increased immune tolerance in EAE (261), decreasing C-reactive protein, IL-6, and TNF-α (262), and improving fatigue in MS (255). Saturated fats have been associated with worse disability outcomes in MS, whereas omega-3 and vitamin D supplementation have been linked to a reduced risk of high-disability trajectories (263). However, many of these studies are limited by small sample sizes and moderate-to-high risk of bias (255). Moreover, the inherent challenge of dietary research of assessing food intake accurately complicates interpretations (258).

Caloric restriction (CR) is another dietary approach that has demonstrated immunomodulatory and neuroprotective effects. In EAE, CR has been shown to suppress age-related immunosenescence by restoring immune cell abundance and enhancing T cell function (264), and to reduce demyelination and neuroinflammation and improve remyelination (265). RCT evaluating CR in pwMS have been very limited, and the few studies available include small samples and younger participants (266). These studies have shown that CR reduces leptin serum levels, increases regulatory T-cell numbers, improves processing information speed, and reduces sNfL in adults with MS (265, 267).

There are no clear guidelines on diet for patients with MS. However, a common characteristic of the abovementioned diets that reduce inflammation or improve outcomes in MS is the prioritization of whole and unprocessed foods. Consumption of ultra-processed food has been associated with immune dysregulation (258), neuroinflammation and cognitive decline (259), acceleration of biological aging (260) and a higher risk of adverse health outcomes (261, 262). In pwMS, higher ultra-processed food consumption has been associated with moderate-to-high MS severity compared to lower consumption (263) and increased likelihood of CNS demyelination (264). To our knowledge, no study has assessed the effect of ultra-processed food in older patients with MS, but similar or worse effects could be expected in this population.

The impact of exercise and dietary modifications in improving biological age in older adults may be increased when both interventions are combined (268). Last but not least, addressing sleep problems in pwMS (269) may mitigate accelerated brain aging (270) and could improve clinical outcomes and QoL in older adults with MS (271, 272).

## 6 Discussion and concluding remarks

As the global population ages, the interaction between aging and MS represents a growing challenge in pathophysiological understanding and clinical management. Older pwMS experience the combined effects of chronic CNS inflammation and progressive neurodegeneration, which are amplified by immunosenescence and inflammaging. These mechanisms are part of the natural history of the disease and may influence therapeutic responses, yet the biological substrates are not yet fully characterized. Although the role of inflammaging in the immunopathogenesis of MS is receiving more attention, most of the available literature centers on conceptual or mechanistic frameworks, but direct evidence associating inflammaging biomarkers, such as pro-inflammatory cytokines, telomere attrition or senescent immune subsets, to clinical or radiological outcomes in MS remains scarce and calls for future prospective studies with biomarkers in older pwMS.

The presence of age-related comorbidities complicates disease monitoring and treatment in older pwMS and present dilemmas regarding de-escalation or discontinuation of DMTs. This highlights the need for multidisciplinary care units to manage these patients. Advances in fluid biomarkers may allow more precise treatment stratification. However, the persistent under-representation of older patients in studies assessing biomarkers and DMTs limits the generalizability of the evidence to this population. Future studies should adopt more inclusive designs and prioritize the characterization of immunosenescence and inflammaging biomarkers. Interventional research combining pharmacological agents with targeted lifestyle modifications may provide new opportunities to mitigate immune aging and preserve neurological function. Together, these strategies may support a move toward therapeutic approaches that put functional outcomes, autonomy, and QoL first in older adults living with MS.
